# Attitudes and Perspectives of People Living With Human Immunodeficiency Virus: Findings From the Positive Perspectives Survey in Slovakia

**DOI:** 10.3389/ijph.2021.642869

**Published:** 2021-10-21

**Authors:** Lubomir Sojak, Katarina Simekova, Lubica Piesecka, Milos Wiesinger, Pavol Jarcuska

**Affiliations:** ^1^ Department of Infectology and Geographical Medicine, Center for Treatment of HIV/AIDS Patients, Academic L. Derer’s University Hospital, Bratislava, Slovakia; ^2^ Department of Infectology and Travel Medicine, Center for Management and Treatment of HIV, University Hospital, Martin, Slovakia; ^3^ Infectology Clinic FSVaZ by UKF, Faculty Hospital, Nitra, Slovakia; ^4^ GlaxoSmithKline, Bratislava, Slovakia; ^5^ Department of Infectology and Travel Medicine, Center for Management and Treatment of HIV, L Pasteur University Hospital and PJ Safarik University, Kosice, Slovakia

**Keywords:** antiretroviral (ARV) therapy, discrimination, human immunodefeciency virus, patient perspectives, Slovakia, stigma

## Abstract

**Objectives:** To investigate the perspectives and attitudes of people living with human immunodeficiency virus (PLHIV) in Slovakia.

**Methods:** A cross-sectional, computer-assisted web survey on health status, emotional support, stigmatisation, communication with physician, treatment, perception, decision-making, concerns, and treatment history. A representative sample of >10% of all PLHIV (*N* = 895) in Slovakia was invited to participate.

**Results:** Mean age of the 117 respondents was 35.4 (±8.9) years, 52.8% had higher education, and 67.0% were in full-time employment. Most (89.4%) were receiving antiretroviral therapy (ART), and 81.8% had undetectable viral load. Most (85.1%) were satisfied with their ART, and side effects were the primary reason for switching therapies. Most (60.8%) had informed only close friends or relatives about their HIV status, only 3 (2.9%) spoke openly about it, and 60.0% hid their ART from others. Of the 31 respondents (31.6%) who experienced stigmatisation, it was primarily from dentists and other physicians who refused to treat them.

**Conclusion:** In general, PLHIV in Slovakia receive ART and are satisfied with it. They do not speak openly about their HIV status, and some have experienced discrimination.

## Introduction

The number of people living with human immunodeficiency virus (HIV) reached 37.7 million globally in 2020, 36.0 million of whom were adults and 1.5 million of whom were new infections [1]. Rates of new HIV diagnoses vary geographically: since 2010, an overall increase in new cases has been observed in the 53 countries of the World Health Organization (WHO) European Region; among the 31 countries of the European Union (EU) and European Economic Area (EEA), new HIV diagnoses decreased by 9% [2]. Geographical variation persists, and within the EU and EEA, some countries have witnessed >100% increases in new diagnoses since 2010, among them Slovakia [2]. In Slovakia, the highest incidence of new HIV cases was reported in 2018 and 2019, with a rate of 1.9 per 100,000 population [2, 3]; despite this increase in HIV diagnoses, Slovakia was among the countries with the lowest average rate of HIV diagnoses in the EU and EEA, which reported overall rates of 5.3 and 4.9 per 100,000 in 2018 and 2019, respectively [2].

In Slovakia, from January 1985 to October 2019, 1160 HIV-positive cases were registered in total [3]. Among these, the infection progressed to acquired immunodeficiency syndrome (AIDS) in 123 persons (110 men, 13 women). During the same period, there were 74 HIV- and AIDS-related deaths [3]. Sexual transmission is the primary route of HIV transmission in Slovakia, occurring in 90% of cases and generally involving men, who compose about 85% of the patient population [3]. Few transmissions were due to needle-sharing (1.8%), even fewer via blood transfusion (0.1%), and none from mother to child [3]. In October 2019, 895 citizens of the Slovak Republic were treated for HIV infection with antiretroviral therapy (ART) [3]. In Slovakia, PLHIV may receive care at any HIV centre, attending visits every 2–3 months to receive ART prescriptions and undergo routine testing according to EUCAST guidelines. Types of ART used in Slovakia may differ from other EU countries because not all drugs are locally available, and some are not reimbursed by insurance companies.

Considerable progress in combating HIV, which has caused 39 million deaths worldwide, has been made with regard to treatment during the last 4 decades [4–6]. With a life expectancy at diagnosis of 1–2 years in the early 1980s to a life expectancy of 53 years if aged 20 years at diagnosis, elimination of HIV appears to be a potentially feasible goal [7, 8]. Such progress has been possible because of the major advancements in ART. To end the AIDS epidemic, however, ART must be made available to all people living with HIV (PLHIV) [4, 5]. Therefore, in 2013, a 90-90-90 goal was set by the United Nations AIDS (UNAIDS) Programme, with an aim of attaining rates of 90% for 1) informing PLHIV that they have HIV to combat underdiagnosis, 2) making ART available to all diagnosed PLHIV, and 3) achieving viral suppression for all PLHIV who are receiving ART by 2020 [4].

Despite this tremendous progress in both treatment efficacy and disease knowledge, PLHIV still suffer from stigmatisation and discrimination and may experience emotional burden due to their illness [9]. Unless these issues are addressed, elimination of HIV will not be possible [9]. Due to stigmatisation and discrimination, PLHIV still suffer a range of abusive behaviours, from physical avoidance to denial of health services and loss of employment [9]. The Positive Voices survey of the life and experiences of PLHIV (N > 4,400), which was conducted in 2017 in the United Kingdom, reported that 11% of survey participants had been refused medical treatment or received it after a delay [10]. One in four suffered anxiety or depressive symptoms daily [10], and 75% needed help dealing with isolation and loneliness [11].

Therefore, we conducted the first survey of PLHIV in Slovakia 1) to evaluate ART use and opinions and expectations regarding ART and 2) to investigate feelings and perspectives amongst PLHIV regarding life after HIV diagnosis, emotional support needs, and experiences with stigmatisation and discrimination.

## Methods

### Survey Design

This was a cross-sectional survey conducted from april to December 2019. Participants completed a computer-assisted web interview (CAWI; Supplementary Material A) consisting of six domains: 1) health status; 2) emotional support; 3) stigmatisation; 4) communication with physician; 5) treatment perception, decision-making, and concerns; and 6) treatment history.

Methodology for questionnaire development has been previously reported [12]. A scientific committee consisting of an HIV clinician, a psychology expert from the Institute of Psychology of Health and Research Methodology (UPZMV) at Medical Faculty of Pavol Jozef Šafárik University (UPJŠ LF) in Košice, Slovakia, and personnel from IQVIA (https://www.iqvia.com/), an internationally recognized contract research organization selected by GlaxoSmithKline to perform the Positive Perspectives survey in Slovakia, contributed to modifying the questionnaire for local use in Slovakia.

Upon agreement to participate, an internet link to the survey was sent to each eligible individual. Respondents were presented with a series of choices. Answering all questions was not compulsory, and respondents were presented with the option to skip questions.

### Survey Population

A nationwide representative sample of HIV-positive people aged over 18 years was selected in cooperation with 5 HIV centres in Slovakia (Supplementary Material B). A sample size of 10% of the total number of 895 ART-treated PLHIV in Slovakia [3] was considered representative and the survey investigators, including HIV clinicians, invited over 100 eligible individuals to participate in the survey.

Those who were eligible to take the survey were HIV-positive individuals aged over 18 years. Eligible PLHIV who had been visiting the 5 HIV centres across the Slovak Republic were quota sampled and then given the details of the study and asked to take the survey. The sampling method was similar to that used in other Positive Perspectives studies [13]. The quotas primarily included the condition of the individual (HIV seropositivity during two independent tests: [1] combined HIV antibodies + *p*24 antigen test, ELISA fourth generation; [2] confirmatory test at Slovakian HIV National Reference Center, Western blot HIV-1/HIV-2 test, and current ART [individual is taking or is scheduled to initiate ART]) to ensure the sample was representative*.*

### Statistical Methods

Data were summarised descriptively; only respondent numbers and percentages for every answer within a given question were calculated. Data were processed and aggregated into computer tabulations and charts and reported by IQVIA.

## Results

### Respondent Characteristics

Overall, 117 PLHIV with a mean age of 35.4 (standard deviation, 8.9) years participated in the survey. Of the 61 survey questions, 70% of questions had ≥80 respondents, 48% had ≥90 respondents, and 30% had ≥100 respondents; only 8% of questions had ≤30 respondents. An average of 6 years had passed since their diagnosis. Most respondents (81.8%) did not have a detectable viral load. Of 49 respondents who provided the location of the HIV/AIDS centre at which they were being treated, most (*n* = 30) were receiving treatment at the University Hospital in Bratislava.

Detailed demographic data and medical history characteristics are summarised in Table 1.

**TABLE 1:** Respondent characteristics at survey completion; Positive Perspectives, Slovakia, 2019.

Responder-reported characteristics, n	Percentage (%) of respondents^a^
*Age groups*	*n = 117*
≤25 years	12.8%
26–30 years	20.5%
31–35 years	22.2%
36–40 years	21.4%
41–50 years	17.1%
>50 years	6.0%
***Gender***	***n = 117***
Male	91.5%
Female	6.8%
Transgender	0.9%
Unknown	0.9%
***Sexual orientation***	***n = 110***
Homosexual/Gay/Lesbian	76.4%
Heterosexual/Orientation to the opposite sex	12.7%
Bisexual	8.2%
Refused to answer	2.7%
***Relationship status***	***n = 110***
Single	42.7%
In relationship and we live together (not married or in a civil partnership)	36.4%
In relationship but not living together	11.8%
Married/Civil partnership	3.6%
Divorced/Separated	2.7%
Refused to answer	2.7%
***Education***	***n = 87***
Elementary	1.1%
Secondary/High school	46.0%
College/University degree	44.8%
Postgraduate/PhD	8.0%
***Employment status***	***n = 88***
Full time	67.0%
Part time	1.1%
Freelancer/Contract work	8.0%
Self-employed full time	1.1%
On disability pension	3.4%
Student	2.3%
Pensioner	1.1%
Volunteer work	1.1%
None of the listed options	9.1%
Refused to answer	5.7%
***Housing***	***n = 88***
I live in my own home	48.9%
I live in a rented home	31.8%
I live in my family home	11.4%
I live with my partner in his/her home	4.5%
I live with friends	2.3%
Refused to answer	1.1%
***Place of residence, type***	***n = 88***
City center	51.1%
City’s suburbs	19.3%
Small town center	13.6%
Small town suburbs	9.1%
Village	6.8%
***Place of residence, name***	***n = 88***
Bratislava region	73.9%
Trnava region	10.2%
Nitra region	5.7%
Trencin region	3.4%
Kosice region	2.3%
Presov region	2.3%
Zilina region	2.3%
***Medical history (diagnoses) other than HIV***	***n = 117***
Sexually transmitted infection	40.2%
Tuberculosis	27.4%
Hypercholesterolaemia	15.4%
Anxiety	13.7%
Gastrointestinal disorders	10.3%
Asthma	9.4%
High blood pressure	8.5%
Insomnia or other sleep disorder	8.5%
Depression or bipolar disorder	6.8%
Heart disease	5.1%
Hepatitis B	5.1%
Drug addiction	4.3%
Hepatitis A	4.3%
Cancer	3.4%
Hepatitis C	3.4%
Schizophrenia	3.4%
Dementia	2.6%
Liver disease	2.6%
Chronic obstructive pulmonary disease	1.7%
Diabetes	1.7%
Kidney disease	1.7%
Osteoarthritis	1.7%
Osteoporosis	1.7%
Epilepsy	1.7%
Vitiligo	0.9%
Posttraumatic stress disorder	0.9%
***Experience with substance use***	***n = 110***
No	60.0%
Yes, occasionally in social events like celebrations and parties	17.3%
Yes, but only marijuana smoking	16.4%
Yes, regularly	6.4%

a Calculated as (number of respondents per given answer*100/the total number of respondents per given question [n]).

n, the number of respondents per given question.

More than half (52.8%) of respondents had a university or college degree, and several had a doctorate degree (Table 1). The majority (67.0%) had full-time jobs. Nearly half (48.9%) owned the home in which they were living, and they resided in the city center (51.1%), most commonly in Bratislava (73.9%; Table 1). From their medical history, the most frequent diagnoses other than HIV were sexually transmitted infections, tuberculosis, hypercholesterolaemia, anxiety, and gastrointestinal disorders (Table 1).

Of the 57 (51.8% of 110; Table 1) who answered that they were in some type of relationship, only 38 gave information related to their partners: 36 (97.4%) said that their partner was aware of their disease, and 25 (65.8%) said that their partner was HIV negative. Of those with HIV-negative partners, 13 were using condoms to prevent HIV transmission during sex, 10 were not using condoms, one was not having sex, and one was using pre-exposure prophylaxis.

### Treatment History and Treatment Perception, Decision-Making, and Concerns

**FIGURE 1:**
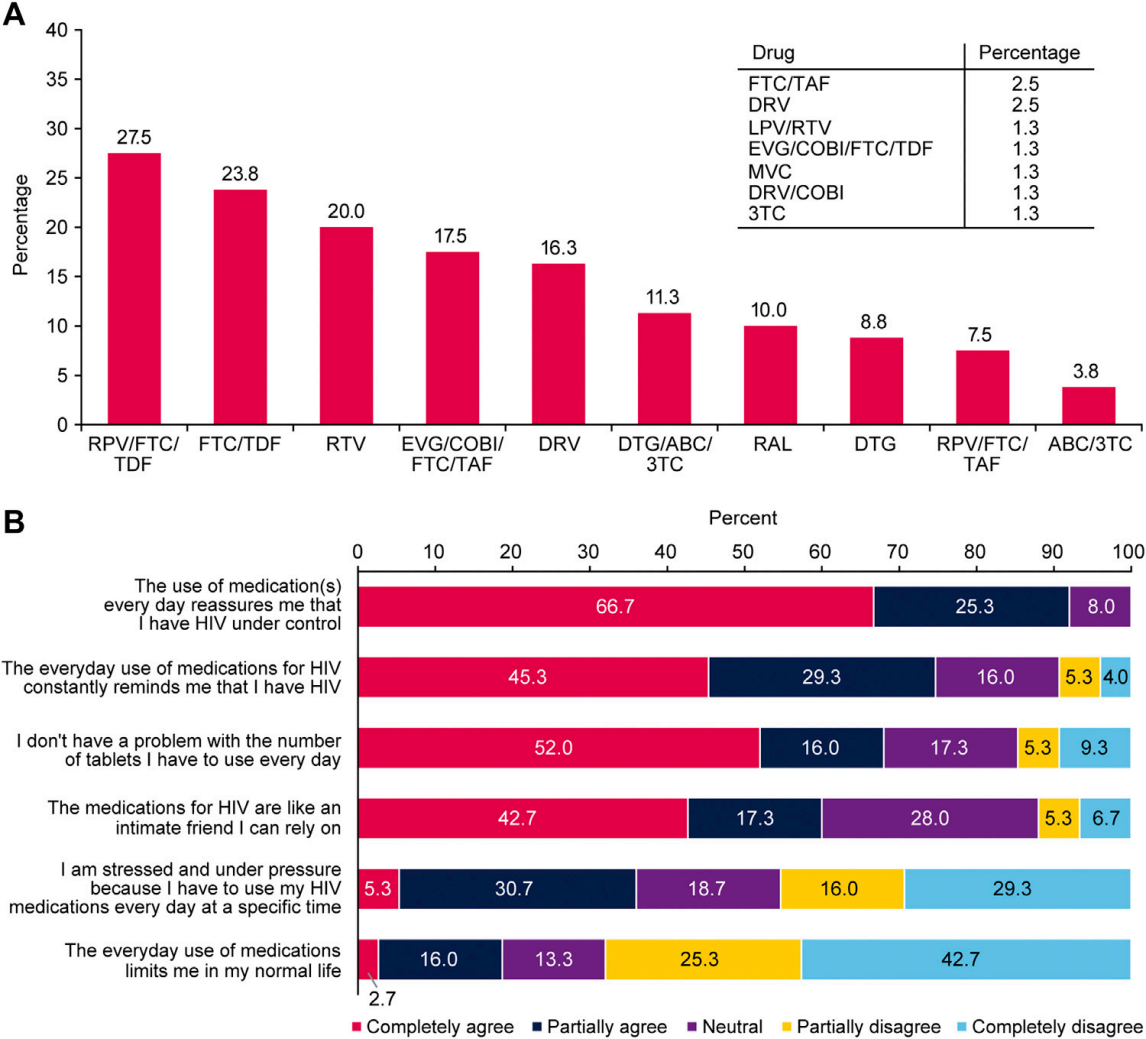
**(A)** Medications currently used for HIV (*n* = 80) and **(B)** respondent feelings about their antiretroviral therapy (*n* = 32); Positive Perspectives, Slovakia, 2019.

Ninety-four participants responded to questions related to their ART. As shown in Table 2, most respondents (84/94, 89.4%) were receiving ART, half as first-line therapy; only 5 (5.3%) were not currently receiving ART, two of whom were newly diagnosed and about to initiate ART. Most respondents (70.0%) had been receiving their current ART regimen for the last 1–4 years, and 85.1% were satisfied with it (Table 2). Among 80 respondents, the most frequently used ART was rilpivirine/emtricitabine/tenofovir disoproxil fumarate (27.5%) and the most frequently used boosting agent was ritonavir (20.0%; Figure 1A). Respondents reported that their medication was keeping their HIV under control (88.8%) and that their health depended on this medication (73.8%; Supplementary Figure S1A). Negative aspects of using medications like adverse effects, or dependence, did not represent a major problem for 38.8% of them (Supplementary Figure S1A). Respondents took, on average, 1.8 tablets of ART daily and 1.7 tablets of another prescribed medication for a different condition. They also took 1 tablet of dietary supplements daily to support their general health. More than half (52.0%, Figure 1B) did not have any problem with the number of daily ART tablets. Most were satisfied or very satisfied with their ART (Table 2). Treatment effectiveness had priority over ART side effects (63.8%; Table 2). Antiretroviral therapy made respondents feel that they had HIV under control (66.7%) but was also a constant reminder of their condition (45.3%; Figure 1B).

**TABLE 2:** Respondent characteristics related to human immunodeficiency virus disease, treatment history, and current status; Positive Perspectives, Slovakia, 2019.

Responder-reported characteristics, n	Percentage (%) of respondents^a^
***Years since HIV diagnosis***	***n = 105***
≤3 years	46.7%
4–5 years	19.0%
6–10 years	16.2%
11–15 years	15.2%
>15 years	2.9%
***At diagnosis, HCP provision of emotional support for HIV diagnosis***	***n = 104***
I was offered/provided counselling and emotional support	44.2%
I was given a recommendation for a patient organisation providing counselling and emotional support	27.9%
I was not offered/provided with any counselling or emotional support, no one offered it to me or talked to me about it	26.9%
I managed it with the help of family and friends	1.0%
***Viral load, levels***	***n = 110***
Not detectable	81.8%
Detectable	7.3%
Unknown	10.9%
***Use of ART***	***n = 94***
Yes, it is my first ART	53.2%
Yes, it is not my first ART	36.2%
Never	5.3%
Not currently	2.1%
Refused to answer	3.2%
***Number of ART tablets received daily***	***n = 80***
1 tablet	55.0%
2 tablets	10.0%
3 tablets	32.5%
4-5 tablets	2.5%
***Dosing of daily ART tablets***	***n = 80***
1 tablet	55.0%
≥2 tablets, altogether once daily	38.8%
≥2 tablets, variable dosing	6.3%
***Duration of current treatment for HIV***	***n = 80***
<1 year	16.3%
1–2 years	37.5%
3–4 years	32.5%
5–6 years	7.5%
>6 years	6.3%
***Level of satisfaction with current treatment for HIV***	***n = 80***
Very satisfied	43.8%
Satisfied	41.3%
Neutral	12.5%
Dissatisfied	2.5%
Very dissatisfied	0%
***Time since last HIV treatment change***	***n = 32***
<6 months	21.9%
7–12 months	15.6%
13–18 months	25.0%
19–24 months	6.3%
>24 months	31.3%
***Decision for HIV treatment change was a decision…***	***n = 32***
Made jointly by the respondent and HCP	37.5%
Made primarily by the HCP	34.5%
Initiated by the respondent	18.8%
Do not remember	6.3%
***The visit to your HCP was …***	***n = 94***
Pleasant	27.7%
Neutral	59.6%
Unpleasant	12.8%
***Preferences on the number of tablets in relation to virus suppression***	***n = 80***
I prefer to reduce the number of ART tablets to the minimum required to suppress the virus	46.3%
I prefer to take the same number of ART tablets to maintain virus suppression	45.0%
I do not know	8.8%
***Preferences on the number of tablets in relation to treatment effectiveness***	***n = 80***
I prefer reducing the drug burden but not at the expense of treatment effectiveness	63.8%
I wonder whether fewer ART tablets would not reduce treatment effectiveness	22.5%
I do not know	13.8%
***Hiding ART from others***	***n = 80***
Yes, always	47.5%
Yes, often	12.5%
Yes, sometimes	32.5%
No, never	7.5%
***Partner’s HIV status***	***n = 38***
Negative	65.8%
Positive	34.2%
Refused to answer	0.0%

a Calculated as (number of respondents per given answer*100/the total number of respondents per given question [n]).

ART, antiretroviral therapy; HCP, healthcare provider; HIV, human immunodeficiency virus; n, the number of respondents per given question.

The choice of changing HIV treatment was made either jointly between respondents and their HIV healthcare provider (HCP; 37.5%) or by the HCP alone (34.5%; Table 2). The main reasons for changing HIV treatment were side effects and achieving better quality of life (QoL; Supplementary Figure S1B). Approximately half (47.9%) of respondents had never discussed their occasional concerns about long-term side effects of HIV medications with their physician, but all had discussed any unwanted adverse effects that involved personal discomfort. In the past 4 weeks, only 6.3% of respondents had been concerned about possible side effects of ART, and in general, they had not reported any increase in urgency regarding concerns relating to ART (Supplementary Figure S1C).

### Communication With Physician

**FIGURE 2:**
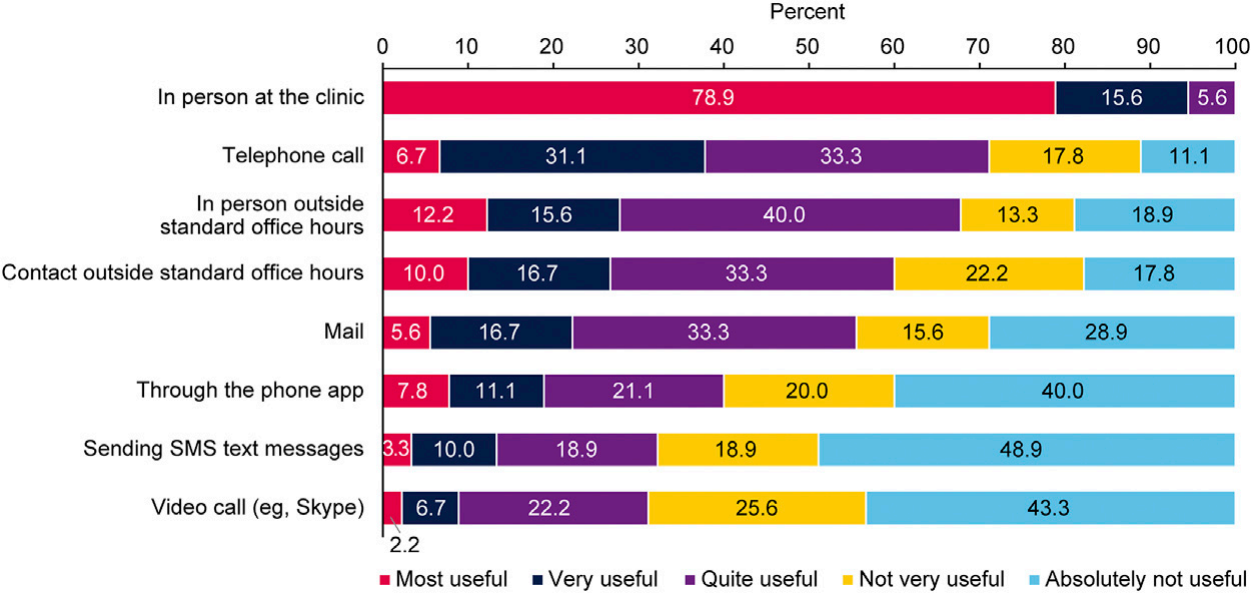
Preferences regarding the type of communication with healthcare providers (*n* = 90); Positive Perspectives, Slovakia, 2019.

Most HCPs were HIV specialists or infectious disease specialists (89/94, 94.7%); only a few were general practitioners (3.2%) or other medical specialists (2.1%). On average, respondents made 4.7 visits per year to their HIV specialist or infectious disease specialist. Respondents preferred personal consultations with their HCP at the clinic and found telephone calls and contact outside standard office hours more useful than SMS messages or video calls (Figure 2).

Most respondents reported that they were actively involved in decision-making regarding their treatment (73.4% for very often and often involved) and were satisfied with this process (74.5% for very often and often; Supplementary Figure S1D). Most respondents reported that they had received sufficient information about HIV treatment (Supplementary Figures S1A,D).

### Health Status and Emotional Support

Emotional support and counselling for dealing with an HIV diagnosis was offered by HCPs to 44.2% of respondents; however, no emotional support was offered or suggested to 26.9% of respondents (Table 2). Immediately after HIV diagnosis, respondents most often sought emotional support from a close friend, on the internet, and from their partner (Supplementary Figure S2). At present, close friends remained the most frequent source of emotional support, followed by partners, another individual with HIV, and the respondent’s HCP (Supplementary Figure S2).

**FIGURE 3:**
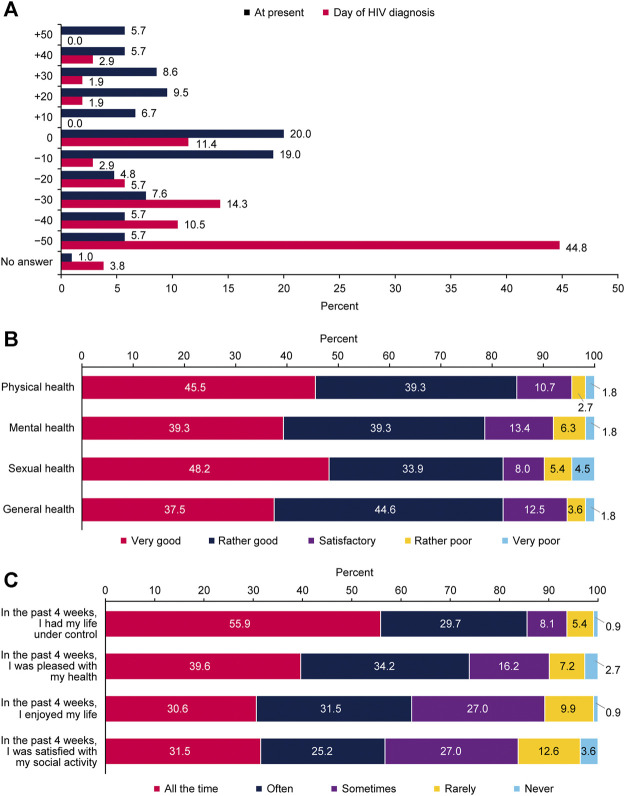
Respondent perspectives on their health and psychological aspects of living with human immunodeficiency virus **(A)** respondent emotions about human immunodeficiency virus diagnosis at the time of diagnosis and at present (*n* = 105) **(B)** evaluation of health status per domain (*n* = 112), and **(C)** respondent satisfaction with life over the past 4 weeks (*n* = 111); Positive Perspectives, Slovakia, 2019.

Respondent ratings on a scale from −50 for most negative emotion to +50 for most positive emotion showed that the day they received their HIV diagnosis was the most negative emotion ever felt for 44.8% (Figure 3A). However, at present, living with HIV was the most negative emotion for only 5.7% of respondents; 20.0% reported that they were indifferent and 25.7% were almost indifferent (−10 and +10 ratings; Figure 3A).

At present, most respondents evaluated their sexual, physical, mental, and general health as very good or rather good (Figure 3B). Always or often during the past 4 weeks, 85.6% of respondents reported that they had their life under control, and 73.8% reported that they were satisfied all the time or often with their health (Figure 3C).

### Stigmatisation

**FIGURE 4:**
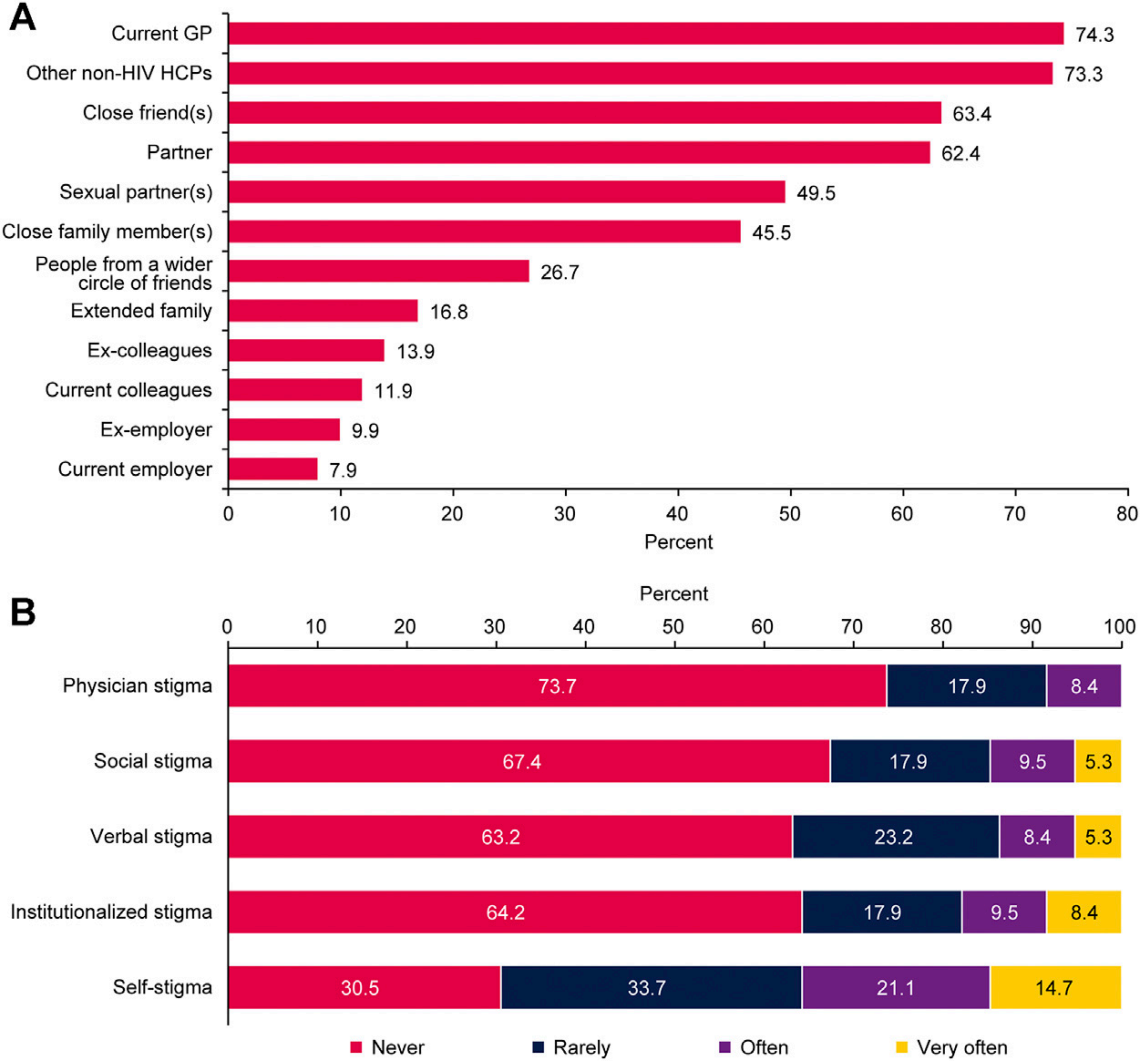
Responses related to the feeling of stigma **(A)** people informed by the respondent about human immunodeficiency virus status (*n* = 101) and **(B)** feeling of stigma due to human immunodeficiency virus status during the past 12 months (*n* = 95); Positive Perspectives, Slovakia, 2019.

The majority of respondents (62/102, 60.8%) said that only very close people knew about their disease and that they would not tell anyone else, 17 (16.7%) said they would speak about their disease if they were asked directly, and only 3 (2.9%) reported talking openly about it. Sixty percent often or always hide their HIV medication from others (Table 2). Most respondents informed other physicians about their condition and told people close to them (friends, partners; Figure 4A). Approximately 10% had informed their employer of their disease (Figure 4A). As recently as within the past 4 weeks, most respondents were afraid of telling other people that they had HIV; fewer were afraid of losing their job if their employer found out they had HIV or of their family finding out (Supplementary Figure S3A). Most respondents said that they had not experienced any type of stigmatisation during the past 12 months (Figure 4B). Of the 31 respondents (31.6%) who had experienced stigmatisation, it was primarily from dentists (11/31, 35.5%) and other HCPs who refused to treat them (6/31, 19.4%; Supplementary Figure S3B).

Respondents reported that educating the general public (59/95, 62.1%) and HCPs (48/95, 50.5%) could reduce feelings of stigma in PLHIV, with most reporting that dentists (39/48, 81.3%) and general practitioners (29/48, 60.4%) were in the greatest need of education on HIV (Supplementary Figure S3C).

## Discussion

This is the first survey on aspects of living with HIV in Slovakian PLHIV. Historically, surveys have played a central role in HIV and AIDS research [14] but often involve only selected aspects of living with HIV. However, the present survey covered a wide range of topics: demographics and disease history; treatment history, preferences, and involvement in decision-making; need for providers of emotional support; experiences with stigmatisation; and communication with HIV HCPs. Overall, we found that PLHIV in Slovakia were generally positive and optimistic about their health and life and satisfied with their current ART, with some experiencing discrimination and stigmatisation.

Respondents to the present survey were generally positive and optimistic about their health and life. Most evaluated their health as good or very good and felt that they had their life under control, with many believing that changing their HIV treatment would improve their QoL. Most survey participants were in full-time employment, highly educated, and could afford living in their home as an owner-occupier or tenant. These characteristics may have contributed to their general positive attitude toward life. A recent survey on QoL in 1462 PLHIV in Spain showed that QoL was positively correlated with education and income [15].

Almost all (89%) respondents were currently receiving ART. Most were satisfied with their current treatment and felt that they were well informed about it. Treatment coverage of about 90% complies with the goal of the UNAIDS Programme for 2020 [4]. Taking medication was a constant reminder of their condition for respondents but at the same time reassured them as they felt they had control over their disease. If they had to change to a different ART, this decision was primarily based on safety issues, and virologic effectiveness was the least frequent reason for changing treatment. These findings may be new to HCPs in Slovakia with regard to preferences of PLHIV on ART. One study showed that although there is general agreement between HCPs and PLHIV on relevant characteristics of ART, the significance of characteristic subcategories is ranked differently by the two groups [16]. Although PLHIV and HCPs ranked the safety profile as the most significant factor in the decision-making process, HCPs thought that some side effects, such as nausea or diarrhoea, were more important to PLHIV than what was reflected in PLHIV’s rankings [16]. Our respondents were receiving an average total number of 4.5 tablets daily, consisting of ART, medication for comorbidities, and dietary supplements. They were not, therefore, considered to be experiencing polypharmacy according to the definitions commonly used in related literature, ie, ≥6 [17] or ≥5 medications daily [18], ≥5 pills or medications for ≥5 health conditions daily [13], and ≥5 non-ART medications daily [19]. This was probably because respondents were relatively young (64% of them aged ≤40 years and only 7 aged >50 years), with low frequencies of comorbidities (Table 1). Nevertheless, these findings, together with respondents’ high ratings of their own health concur with evidence showing that polypharmacy is associated with reduced feelings of well-being and low ratings of satisfaction with physical, mental, and sexual health [13].

An important finding from the present survey is that respondents mostly felt that they were involved in decision-making about their treatment and were satisfied with the process. Respondents unequivocally preferred face-to-face communication at the clinic; they perceived telephone calls or contact outside standard office hours as useful. They appeared to make little use of SMS text messages and Skype. Yelverton et al [16] also reported that open and good communication with HCPs was important for PLHIV. However, these findings were made before the recent coronavirus pandemic forced people around the globe to adopt new technology for virtual meetings and discussions as a safety precaution to lower the risk of contracting COVID-19, which is particularly important for at-risk populations such as PLHIV.

With respect to emotional support, receiving their HIV diagnosis was the most negative emotion that almost half of respondents could ever imagine. This was also the case in a large survey conducted among 1111 PLHIV in nine countries: Australia, Austria, Canada, France, Germany, Italy, Spain, United Kingdom, and United States [20]. Similar also were the rates of participants who were offered emotional support or were referred for counselling by their primary HCP at diagnosis: 68% in the overall population, ranging from 52% in Spain to 84% in Australia, and 72.1% in our survey (Table 2). It is alarming, however, that so many PLHIV were not offered any emotional support from their HIV specialist at diagnosis (Table 2). The reasons for this must be thoroughly researched, and corrective measures should be implemented. Close friends and partners were the main sources of emotional support for our respondents. A minority of respondents gave information on their partners, and they mostly reported that their partners were HIV negative and that more than half used condoms. This means that about 40% were still having unprotected sex, indicating that there is still a need for education on prevention of infection.

Although most respondents did occasionally hide their HIV medications, many had not experienced any form of stigmatisation during the past year, contrary to previous evidence [20]. Only few respondents would openly talk about their HIV diagnosis, contrary to a European average of 21%, an Australian average of 33%, and an even higher average of 42% in North America (US and Canada) [20]. Few respondents had informed their colleagues and employers about their HIV diagnosis, an even smaller proportion than the lowest respective percentage reported by the Italian branch of the Positive Perspectives survey (24%) [20]. These findings may suggest that PLHIV in Slovakia prefer to proactively protect themselves from negative attitudes and behaviours based on prejudice against those with HIV [21]. Regarding whom they were afraid to inform about their HIV diagnosis, respondents were most concerned about telling other people they have HIV and least concerned about their family and losing their job. During the past year, 1 out of 3 respondents had experienced discrimination, often because their dentist had refused to treat them. Stigmatisation is not new for PLHIV who have long suffered such behaviour in their everyday lives and—worse—in other settings, primarily dental care [10]. In a United States survey, PLHIV reported having suffered a range of concerns and fears, either anticipating discrimination by their dentist or disrespectful behaviour during an actual visit [22]. To protect themselves from such discrimination, they selected dentists with known experience in treating PLHIV, as shown in a Canadian survey [23]. In the United Kingdom, the 2017 Positive Voices survey reported that 11% of the 4400 PLHIV surveyed had been denied provision of or had received belated care, 14% had experienced some sort of discrimination, and 18% avoided seeking care when needed [10]. In the 2015 Stigma survey, discriminatory experiences were reported in the dental setting (15%) and among general practitioners (13%) [24]. Participants in the Canadian survey also reported that discriminatory behaviour had decreased over the years after educational interventions, clinical experience, and inclusion of HIV content in dental school curricula [23]. A study from the Pacific AIDS Education and Training Center in the United States showed that HIV-related knowledge, beliefs, and behaviours of dentists significantly improved after an HIV-related educational program [25]. Our respondent subgroup who reported having experienced stigmatisation also suggested that educational programs should be designed for dentists, other HCPs, and the general public. It is evident that this request should be fulfilled to ensure equal treatment of all individuals, and PLHIV in Slovakia should feel confident and safe using the healthcare system [10]. This finding highlights the need to educate non-HIV HCPs on all aspects of living with HIV and AIDS. Dentists and general practitioners in particular show a deficit of knowledge, as well as non-HIV and medical nurses. To help address the issue of HIV stigmatisation in healthcare settings, HIV awareness education symposia and lectures for general practitioners and nurses in Slovakia were conducted in 2020 [26, 27]. These presentations provided general practitioners and nurses with general HIV disease education and emphasized that the risk of HIV transmission to healthcare providers is very low. Similar lectures on HIV awareness education for dentists are planned.

### Limitations

As this was a convenience-sample survey, it is not generalisable to people who do not wish to answer survey questions or give information about their experience with living with HIV or AIDS. Furthermore, because it was an online survey, the findings may not be generalisable to PLHIV who are not familiar with or do not wish to participate in online surveys. However, because these findings are based on responses from more than 10% of the ART-treated population of PLHIV in Slovakia [3], it is likely we included a representative sample. A further limitation of our survey is that we included a smaller percentage of women (6.8% in the survey vs 10.8% in the total Slovakian population with HIV in 2019), and we did not explore associations between participant characteristics and treatment preferences, experiences of stigmatisation, and other factors.

### Conclusion

In Slovakia, PLHIV are often well educated and employed full time. They are positive and optimistic about their health and life, as most evaluated their health as good or very good and reported having their life under control. They receive ART and are satisfied with it. They generally do not speak openly about their HIV status, and some have experienced discrimination and stigmatisation. To improve the issue of HIV stigmatisation in healthcare settings, we recommend that healthcare providers, including general practitioners and dentists, attend symposia and lectures to receive education on all aspects of living with HIV and AIDS. Our results will be of interest to physicians wishing to increase their understanding of the issues facing PLHIV and their attitudes on ART. Policymakers should consider our findings when designing and implementing necessary guidelines and educational programs that aim to eliminate stigmatisation and discrimination against PLHIV.

## Data Availability

The original contributions presented in the study are included in the article/Supplementary Material, further inquiries can be directed to the corresponding author.

## References

[1] UNAIDS. Fact Sheet: Global HIV Statistics (2021). Available at: https://www.unaids.org/sites/default/files/media_asset/UNAIDS_FactSheet_en.pdf. (Accessed July 30, 2021).

[2] European Centre for Disease Prevention and Control. HIV./AIDS Surveillance in Europe 2020: 2019 Data (2020). Available at: https://www.ecdc.europa.eu/sites/default/files/documents/hiv-surveillance-report-2020.pdf. (Accessed July 30, 2021).

[3] Public Health Office of the Slovak Republic. Úrad verejného zdravotníctva slovenskej republiky. Výskyt infekcie hiv v slovenskej republike k 31.10.2019 (occurrence of hiv infection in the slovak republic as of 31.10.2019) (2019). Available at: http://www.uvzsr.sk/docs/info/epida/hiv_k31okt2019.pdf. (Accessed August 14, 2020).

[4] UNAIDS. 90-90-90: an Ambitious Treatment Target to Help End the AIDS Epidemic (2017). Available at: https://www.unaids.org/en/resources/909090 (Accessed August 15, 2020).

[5] Fauci AS Marston HD. Ending the HIV-AIDS Pandemic - Follow the Science. N Engl J Med (2015) 373:2197–9. 10.1056/nejmp1502020 26624554

[6] Sharp PM Hahn BH. Origins of HIV and the AIDS Pandemic. Cold Spring Harbor Perspect Med (2011) 1:a006841. 10.1101/cshperspect.a006841 PMC3234451 22229120

[7] Fauci AS Folkers GK Marston HD. Ending the Global HIV/AIDS Pandemic: the Critical Role of an HIV Vaccine. Clin Infect Dis (2014) 59;(Suppl. 2):S80–4. 10.1093/cid/ciu420 25151483 PMC4157695

[8] Marcus JL Chao CR Leyden WA Xu L Quesenberry CP Jr Klein DB Narrowing the gap in Life Expectancy between HIV-Infected and HIV-Uninfected Individuals with Access to Care. J Acquir Immune Defic Syndr (2016) 73:39–46. 10.1097/qai.0000000000001014 27028501 PMC5427712

[9] UNAIDS. Evidence for Eliminating HIV-Related Stigma and Discrimination: Guidance for Countries to Implement Effective Programmes to Eliminate HIV-Related Stigma and Discrimination in Six Settings (2020). Available at: https://www.unaids.org/sites/default/files/media_asset/eliminating-discrimination-guidance_en.pdf. (Accessed August 13, 2020).

[10] National AIDS Trust. Changing Perceptions: Talking about HIV and Attitudes (2018). Available at: https://www.nat.org.uk/sites/default/files/publications/web_PV_Changing%20Perceptions-Stigma-report.pdf. (Accessed August 13, 2020).

[11] National AIDS Trust. Changing Perceptions: Talking about HIV and Our Needs (2018). Available at: https://www.nat.org.uk/sites/default/files/publications/web_PV_Changing%20Perceptions-needs-report.pdf. (Accessed August 13, 2020).

[12] de los Rios P Okoli C Young B Allan B Castellanos E Brough G Treatment Aspirations and Attitudes towards Innovative Medications Among People Living with HIV in 25 Countries. Popul Med (2020) 2:23. 10.18332/popmed/124781

[13] Okoli C de Los Rios P Eremin A Brough G Young B Short D. Relationship between Polypharmacy and Quality of Life Among People in 24 Countries Living with HIV. Prev Chronic Dis (2020) 17:E22. 10.5888/pcd17.190359 32134717 PMC7085909

[14] National Research Council. Methodological Issues in AIDS Surveys. In: National Research CouncilEvaluating AIDS Prevention Programs. Expanded Edition. Washington, DC: National Academies Press (1991). p. 207–316.

[15] Fuster-RuizdeApodaca MJ Laguía A Safreed-Harmon K Lazarus JV Cenoz S Del Amo J. Assessing Quality of Life in People with HIV in Spain: Psychometric Testing of the Spanish Version of WHOQOL-HIV-BREF. Health Qual Life Outcomes (2019) 17:144. 10.1186/s12955-019-1208-8 31426799 PMC6700970

[16] Yelverton V Ostermann J Hobbie A Madut D Thielman N. A Mixed Methods Approach to Understanding Antiretroviral Treatment Preferences: what Do Patients Really Want. AIDS Patient Care and STDs (2018) 32:340–8. 10.1089/apc.2018.0099 30179532 PMC6755657

[17] Gleason LJ Luque AE Shah K. Polypharmacy in the HIV-Infected Older Adult Population. Clin Interv Aging (2013) 8:749–63. 10.2147/CIA.S37738 23818773 PMC3693722

[18] Edelman EJ Gordon KS Glover J McNicholl IR Fiellin DA Justice AC. The Next Therapeutic challenge in HIV: Polypharmacy. Drugs Aging (2013) 30:613–28. 10.1007/s40266-013-0093-9 23740523 PMC3715685

[19] Siefried KJ Mao L Cysique LA Rule J Giles ML Smith DE Concomitant Medication Polypharmacy, Interactions and Imperfect Adherence Are Common in Australian Adults on Suppressive Antiretroviral Therapy. AIDS (2018) 32:35–48. 10.1097/qad.0000000000001685 29135584 PMC5732638

[20] ViiV Healthcare. The Positive Perspectives Survey Report: a View into the Lives of People Living with HIV (2017). Available at: https://edgesuite.gskstatic.com/Viiv/viivhealthcare/pdf_files/master/main/positive-perspectives-survey-report-finalcompressed.pdf. (Accessed November 10, 2020).

[21] Centers for Disease Control and Prevention. HIV Stigma and Discrimination (2020). Available at: https://www.cdc.gov/hiv/basics/hiv-stigma/index.html. (Accessed August 12, 2020).

[22] Patel N Furin JJ Willenberg DJ Apollon Chirouze NJ Vernon LT. HIV-related Stigma in the Dental Setting: a Qualitative Study. Spec Care Dentist (2015) 35:22–8. 10.1111/scd.12078 25039662 PMC4785592

[23] Brondani MA Phillips JC Kerston RP Moniri NR. Stigma Around HIV in Dental Care: Patients' Experiences. J Can Dent Assoc (2016) 82:g1. 27548661

[24] Okala S Doughty J Watt RG Santella AJ Conway DI Crenna-Jennings W The People Living with HIV STIGMASurvey UK 2015: Stigmatising Experiences and Dental Care. Br Dent J (2018) 225:143–50. 10.1038/sj.bdj.2018.530 30050184

[25] Mulligan R Seirawan H Galligan J Lemme S. The Effect of an HIV/AIDS Educational Program on the Knowledge, Attitudes, and Behaviors of Dental Professionals. J Dental Edu (2006) 70:857–68. 10.1002/j.0022-0337.2006.70.8.tb04152.x 16899445

[26] Šimeková K. HIV Infection in a General Practitioner's Office. In Presented at: 41st Annual SSVPL Conference. Slovakia: Vysoké Tatry (2020).

[27] Balogová L. Shared Care for Patients with HIV/AIDS. In Presented at: 41st Annual SSVPL Conference. Slovakia: Vysoké Tatry (2020).

