# Supplementary material for: Attitudes and Perspectives of People Living With Human Immunodeficiency Virus: Findings From the Positive Perspectives Survey in Slovakia
Source: Int J Public Health. 2021 Oct 21;66:642869. doi: 10.3389/ijph.2021.642869 (PMC8565261; doi:10.3389/ijph.2021.642869)

Supplementary material

A. Survey questionnaire

A01: Indicate the gender you identify with

- Male
- Female
- Transsexual - woman to man
- No answer

A02: Age of patient

- Up to 25 years
- 26-30 years
- 31-35 years
- 36-40 years
- 41-50 years
- Over 50 years

A03: Have you ever been diagnosed with any of the following diseases by your physician or any healthcare provider?

- HIV (human immunodeficiency virus)
- Sexually transmitted infection (apart from HIV alone, eg, HPV, chlamydia, gonorrhea, syphilis)
- TB (tuberculosis)
- High cholesterol (hypercholesterolaemia)
- Anxiety
- Gastrointestinal tract disorders (eg, peptic ulcer, duodenal ulcer, gastroesophageal reflux, ulcerative colitis, Crohn's disease, irritable bowel syndrome)
- Asthma
- High blood pressure (hypertension)
- Insomnia or other sleep disorder
- Depression/Bipolar disorder
- Heart disease (eg, cardiomyopathy, coronary artery disease, stroke, heart failure, irregular pulse)

- Hepatitis B
- Drug addiction
- Hepatitis A
- Cancer
- Hepatitis C
- Schizophrenia
- Dementia
- Liver disease (eg, hepatic steatosis, cirrhosis)
- COPD (chronic obstructive pulmonary disease)
- Diabetes mellitus
- Kidney disease (eg, acute renal failure, chronic kidney disease)
- Osteoarthritis
- Osteoporosis
- Epilepsy
- Vitiligo
- Posttraumatic stress disorder

A04: How would you define your sexual orientation?

- Homosexual/Gay/Lesbian
- Heterosexual/Orientation to the opposite sex
- Bisexual
- Refusal to answer

A05: Which of the following options best describes your status in terms of relationships?

- Single
- In a relationship and we live together (not married or in a civil partnership)
- In a relationship but not living together
- Married/Civil partnership
- Divorced/Separated
- Refusal to answer

A06: How many years ago were you diagnosed HIV positive?

- o 3 years
- o 4-5 years
- o 6-10 years
- o 11-15 years
- o Over 15 years

A07: Is your viral load currently undetectable?

- o Yes
- o I don´t know
- o No

A08: Do you have any personal experience with drug use in the last 12 months (eg, marijuana, cocaine, ecstasy, methamphetamine, heroin)?

- o No
- o Yes, occasionally (eg, during celebrations, parties, social events)
- o Yes, but only smoking marijuana
- o Yes, regularly

B01: How would you describe your health (whether or not related to HIV) in the last 6 months? Evaluation for each category as very good = 1; rather good = 2; satisfactory = 3; rather poor = 4; and very poor = 5

- o Physical health
- o Mental health
- o Sexual health
- o Overall health

B02: Select one answer to each statement regarding your satisfaction with life in the past 4 weeks:

- o In the past 4 weeks, I had my life under control
- o In the past 4 weeks, I was pleased with my health
- o In the past 4 weeks, I enjoyed my life
- o In the past 4 weeks, I was satisfied with my social activity

C02: To what extent do you agree or disagree with the following statements?

- I believe that advances in HIV treatment will improve the quality of my life
- Since being diagnosed HIV positive, my life has taken on a different meaning
- My aspiration related to work/career has not changed because of HIV
- Although it is possible to keep HIV under control, I am concerned about its effect on my sex life
- Because of HIV, I have made positive changes to my life, which I might not have ever made otherwise
- I think HIV will cut my life short
- Thanks to HIV, it is more likely that I will live my life to the full every day
- The fact that I am HIV positive helped me create really meaningful relationships
- I don't think that HIV limits me in my life anyhow
- Because of HIV, I don't make any plans for the distant future

D01a: How would you describe the effect of the confirmation that you are HIV positive on your emotions?

[Express on a scale ranging from −50 to +50, where +50 is the most positive effect on emotions you can imagine, 0 means no change in your emotional state compared with how you felt before HIV diagnosis, and −50 means the most negative effect on emotions you can imagine.]

- −50, the most negative effect on emotions
- −40
- −30
- −20
- −10
- 0, no change in your emotional state
- +20
- +30
- +40
- +50, the most positive effect on emotions
- Refusal to answer

D01b: How would you describe your emotional life with HIV today?

[Express on a scale ranging from −50 to +50, where +50 is the most positive effect on emotions you can imagine and 0 means no change in your emotional state compared with how you felt before the determination]

- −50, the most negative effect on emotions
- −40
- −30
- −20
- −10
- 0, no change in your emotional state
- +20
- +30
- +40
- +50, the most positive effect on emotions
- Refusal to answer

D02: Which of the following statements best describes what happened after initial diagnosis of HIV - what, if any, emotional support or counselling was provided by your treating physician?

- Was offered/provided counselling and emotional support
- Was provided a referral to a patient organisation providing counselling and emotional support
- Was not offered/provided with any counselling or emotional support; no one offered it to me, and no one talked to me about it, either
- Coped with it myself and with the help of my family and friends

D03: Where did you look for emotional support immediately after you were diagnosed with HIV?

- From my close friend
- On the internet and online discussion forums
- From my partner, my husband/wife, my other half
- From someone who also has HIV
- From the treating physician/medical nurse
- From an immediate member of the family (eg, siblings, parents)
- Nowhere, I didn’t seek help
- On social media
- In the organisation providing help to patients

- From the psychologist
- In personal meetings with a group of people who also have HIV
- At my GP

D04: From whom would you prefer to receive emotional support in relation to your current needs?

- From my close friend
- From someone who also has HIV
- From my partner, my husband/wife, my other half
- From the treating physician/medical nurse
- From an immediate member of the family (eg, siblings, parents)
- At an organisation providing help to patients with HIV
- At face-to-face meetings with a group of people who also have HIV
- From no one, I don’t need any support
- From the psychologist
- On the internet and online discussion forums
- From my general practitioner
- On social media
- Generally, from people around

D05: Does your partner know about your HIV infection?

- Yes
- Refusal to answer

D06: How does your partner support you in relation to HIV?

- Provides emotional support, is someone I can talk to about HIV
- Reminds me to take HIV medications
- Reminds me the check-up dates at the doctor’s
- Helps me with preparing medications for use
- Goes with me to regular medical check-ups but is not present during the consultation
- Goes with me to regular medical check-ups and is present during the consultation
- Helps me open the discussion about what worries me with my physician

D07: What is the HIV status of your partner?

- HIV negative
- HIV positive

D08: How do you protect yourself to prevent HIV transmission to your partner?

- We use condoms when having sex
- No protection
- We are not having sex
- We use PrEP

E01: Which of the following options best describes your level of openness in relation to your HIV?

- Only very close people know about my HIV status and I would not tell anyone even if they asked me directly
- None of the listed options
- If asked a direct question, I would openly answer, however, I feel no need to tell everyone
- Generally, I openly talk about my HIV status

E02: Who, except your physician, knows about your HIV status? Who did you inform about it?

- My current general practitioner
- One or more other healthcare providers (eg, medical nurses, counsellors, pharmacists, psychiatrists)
- One or more close friends
- Partner
- One or more sexual partners
- One or more members of the immediate family (excluding partner, husband/wife)
- One or more people from a wider circle of friends
- One or more members of the wider family
- One or more colleagues in my previous workplace
- One or more colleagues in my current workplace
- My previous employer
- My current employer

E03: Select one answer for each of the statements regarding your concerns about the revelation of your HIV

infection in the past 4 weeks.

- In the past 4 weeks, I was afraid of telling other people that I have HIV
- In the past 4 weeks, I withheld information I tell about myself to other people
- In the past 4 weeks, I was afraid that my colleagues would find out I have HIV
- In the past 4 weeks, I was afraid that if they find out I have HIV, I'll lose my job
- In the past 4 weeks, I was afraid that the members of my family would find out that I have HIV

E04a: In the past 12 months, did you have a feeling of stigmatisation?

- No
- Yes

E04b: Could you describe specific examples having happened in the past 12 months when you personally experienced stigmatisation due to HIV?

- Refused dental treatment
- Refused medical treatment (general)
- Unpleasant behaviour/Disrespect of the physician
- Negative opinions of colleagues
- Concerns of my partner about disease transmission and ending the relationship
- Lack of comprehension of people that it is a disease
- Opinion that sexual diseases are associated with a gay sexual orientation
- Contempt of my close friends
- False information about HIV even among open people
- People loathe me
- Refused sex
- Opinion that people themselves are to be blamed for HIV
- Blackmailing to reveal my diagnosis to close people around me

E05: How often, if at all, did you feel each of the following types of stigma in the past 12 months?

- Physical stigma
- Social stigma
- Verbal stigma

- Institutionalised stigma
- Self-stigma

E06: Which of the following options could, in your opinion, reduce feelings of stigmatisation in people with HIV?

- Education of the general public
- Education of healthcare providers
- Education in schools
- Legislation with the aim to limit the level of discrimination
- Higher social responsibility and education through the media
- Increased awareness through social media, discussion forums with the aim to educate/fight against stigma
- Increased activity among patient organisations with the aim to educate/fight against stigma
- Don’t know
- None of the listed options, can’t see any need for it

E07: Which healthcare providers would, in your opinion, particularly need education in the area of HIV to help reduce the feeling of stigmatisation?

- Dentists
- General practitioners
- Other specialists (not HIV)
- Medical nurses
- Medical nurses in hospitals
- Dermatologists
- Social workers
- Gynecologists
- HIV specialists
- Pharmacists
- Don’t know

F01: How many times in the past 12 months, if at all, have you visited the following healthcare providers in relation to HIV?

- HIV specialist or infectologist

- Another specialist (ie, not HIV/infectious disease)
- Other (centre for assisted reproduction, dermatologist, ENT, psychiatrist, dentist, urologist, institution for drug addiction)
- General practitioner

F02: Which type of healthcare providers do you see as your main healthcare provider for HIV treatment?

- HIV specialist or infectologist
- General practitioner
- Specialist in different field (ie, not HIV/infectious disease)

F03a: To what extent is it generally pleasant to you to discuss HIV and problems that bother you with your physician?

- Pleasant
- Neutral
- Unpleasant

F03b: Why was it not pleasant for you to discuss HIV and problems that bother you with your physician?

- It seems that my physician always knows everything best
- None of the options - it was always very pleasant to me
- I don’t know exactly how to start talking about it
- I don’t think it is that important to trouble him
- I don’t want to keep him any longer
- I don’t believe he could sort out my problem
- I don’t know
- It seems like there is never time or the chance to do so

F04: To what extent do you find the following forms of communication with your HIV specialist useful (or would, if available)?

- In person at the clinic
- Telephone call
- In person at a place where medical care is not provided
- In person outside standard office hours

- Mail
- Through the phone app
- Sending SMS text messages
- Video call (eg, Skype)

F05: You have mentioned telling your general practitioner that you have HIV. What made you do so?

- I wanted the GP to be involved and informed about my health status in relation to HIV
- I wanted the GP to know which HIV medications I use in case this would be a problem in connection with other prescription drugs
- My treating physician told me it would be best if I did so
- I didn’t have a choice in the matter as he was informed by my treating physician
- My general practitioner diagnosed me with HIV
- I needed a document about periodical medical examinations for my employer
- It is mandatory

F06: You have mentioned not telling your general practitioner that you have HIV. Why?

- If not necessary, I’d rather not tell people about it
- I want to separate general health problems and HIV treatment
- I am concerned about his reaction, that he might perceive me differently or my relationship with him would change
- I am concerned I would have to change my general practitioner
- I am concerned he might not have the knowledge/education to deal with HIV-positive people
- I don’t think it is necessary
- After being diagnosed, I have not visited the general practitioner yet

G01: Select one answer to each statement regarding your involvement in the choice of your medications.

- The physician asks me how I tolerate the treatment
- The physician would always explain to me why he wants me to use that medication
- I think I am actively involved in the decision-making about my treatment
- I am satisfied with my involvement in the decisions about my treatment
- The physician making the choice of treatment considers my subjective view of the matter

- The physician will explain the various treatment options but will leave the decision how to proceed to me
- My physician decides and selects the medication for me irrespective of my preferences
- The physician supports me in my decisions, regardless of whether he agrees with them or not
- I don’t have enough information to be actively involved in the decision-making about the treatment
- I am the one to decide whether I will start or stop using any medications and not my physician

G02a: Do you currently use antiretrovirals for treatment of HIV?

- Yes, it is my first antiretroviral
- Yes, but it is not my first antiretroviral
- No, I have never used antiretrovirals
- Refusal to answer
- No, I don’t use antiretrovirals but used them in the past

G02b: For what reasons did you stop using the antiretroviral drug for treatment of HIV?

- My physician recommended me not to use medication at this phase
- It caused complications because I had to use medications for other conditions as well

G02c: For what reasons did you not start using any antiretroviral drug for treatment of HIV?

- I have been diagnosed only recently and plan to start treatment soon
- I am concerned about the effect of long-term treatment on my body
- I am concerned about cost; I can’t afford it
- I can’t see any need to start with treatment when I feel healthy
- I am concerned about the possibility that if I start treatment now, I will become resistant to it over time

G03: To what extent do you agree or disagree with each of the following statements in relation to HIV medications?

- These medications keep HIV under control
- My health depends on these medications
- My health in the future will depend on these medications
- Without these medications, I would be very ill
- I have received enough information about HIV treatment
- My life without these medications would not be possible
- These medications keep me alive

- These medications are my only hope for the future
- Sometimes I am concerned about the long-term effects of these medications
- If I miss a day, it will not cause problems in the long run
- It is unlikely that I will experience any unpleasant side effects from this treatment in the next month
- The need to use the medication worries me
- These medications are a mystery to me
- These medications disrupt my life
- Sometimes I'm worried I will become addicted to these medications
- The use of these medications is shameful
- These medications cause unpleasant side effects to me
- Use of these medications is far worse than I expected
- The taste of these medications makes me sick

G04: You mentioned you are sometimes concerned about long-term effects of medications for HIV. Have you ever discussed these concerns with your physician?

- No
- Yes
- Can't recall

G05: You mentioned that medications for HIV sometimes cause unpleasant side effects to you. Have you ever discussed these side effects with your physician?

- Yes, I usually start to discuss it during the visit
- Yes, sometimes it is me who starts to discuss it and sometimes it is the physician who asks me about it

G06: Select one answer for each of the statements regarding your feelings about the use of HIV medications in the past 4 weeks.

- I was concerned about possible side effects of the medication on my body
- They were a burden to use
- The use of medication caused me unpleasant side effects
- Because of the use of medication, it was difficult for me to lead a normal life
- I was not sure why I use the medication

G07: Which medication(s) for HIV do you currently use?

G08: To what extent are you satisfied with your current HIV treatment?

- Very satisfied
- Satisfied
- Neither satisfied nor dissatisfied
- Dissatisfied
- Very dissatisfied

G09: How long have you been using your current medications for treatment of HIV ?

- Less than 1 year
- 1-2 years
- 3-4 years
- 5-6 years
- More than 6 years

G10: Do you have to hide your medications for HIV so that other people will not find out that you have HIV?

- Yes, all the time
- Yes, often
- Yes, sometimes
- No, never

G11: How many tablets from each category do you use daily?

- HIV (include only prescription drugs)
- Vitamins, food supplements, herbal preparations, etc
- Other diseases (include only prescription drugs)

G12: How often do you use medications for HIV?

- 1 tablet, once daily
- 2 or more tablets, all once daily
- 2 or more tablets, some once daily, others twice daily

G13: For what reasons do you use vitamins, mineral supplements, or herbal preparations?

- To boost general health

- I am concerned that I don’t get vitamins/minerals I need from my diet
- As a supporting product in the treatment of other diseases or problems not related to HIV
- As a supporting product against fatigue/exhaustion
- To suppress the effect of antiretrovirals on my body

G14: When was the last time that your HIV treatment was changed?

- 0-6 months ago
- 7-12 months ago
- 13-18 months ago
- 19-24 months ago
- More than 24 months ago

G15: Who decided to change your HIV treatment?

- It was a common decision of mine and my physician
- The change was primarily the decision of my physician
- The change was initiated by me and it was my decision
- I don’t recall

G16: Which of the following reasons is the most important factor driving the decision about the change of your HIV treatment?

- To reduce the severity or frequency of side effects
- To improve my quality of life
- To reduce the number of tablets I have to take
- To reduce the frequency of dosing (eg, from twice daily to once daily)
- To reduce the number of medications used in the overall treatment of HIV
- To reduce the cost of medications
- I can’t recall
- My previous medications didn’t sufficiently control my viral load, or I was becoming resistant to them
- To limit potential drug interactions with the other medications I use/used
- To make it possible for me to use with food/without food
- To improve compliance (my ability to use medications according to the instructions for use)

- Found lactose intolerance - switch to a medication without lactose
- Switch to a milder form of the medication

G18: Based on your experience with the treatment of HIV, to what extent do you agree or disagree with each of the following statements?

- The use of medication(s) every day reassures me that I have HIV under control
- The everyday use of medications for HIV constantly reminds me that I have HIV
- I don’t have a problem with the number of tablets I have to use every day
- The medications for HIV are like an intimate friend I can rely on
- I am stressed and under pressure because I have to use my HIV medications every day at a specific time
- The everyday use of medications limits me in my normal life

G23: Select the statement that best describes your opinion of the number of different HIV medications used in your treatment with antiretrovirals.

A) Number of HIV medications and virus suppression
   - I prefer to reduce the number of medications for HIV I use to the minimum needed to suppress the virus
   - I prefer to use the same number of medications for HIV to keep the virus suppressed
   - I don’t know

B) Number of HIV medications and effectiveness of treatment
   - I prefer to reduce the burden of medicines (polypharmacy) if it doesn’t mean a less effective treatment
   - It would worry me if a lower number of HIV medications in my regimen won’t reduce the effectiveness of treatment
   - I don’t know

C) Number of HIV medications and switch to a new treatment
   - If the virus remains suppressed in my body, I am open to switch to the HIV treatment that includes a lower number of medications
   - If the virus remains suppressed in my body and I am satisfied with the medications, I would not consider switching to a new treatment

- I don’t know

D) Switch to the injectable treatment

- If the virus remains suppressed in my body, I am open to use the injectable treatment once every 2 months
- If the virus remains suppressed in my body and I am satisfied with the form of treatment, I would not consider the option to use the injectable treatment once every 2 months
- I don’t know

H01: What is your highest level of education finished?

- Elementary school
- Secondary/High school
- College/University degree
- Postgraduate/PhD

H03: Which of the following options best describes your work status?

- Full-time employment
- None of the listed options
- Freelancer/Based on a contract
- Refusal to answer
- On disability pension
- Student, regular daily study
- Volunteer
- Old-age pensioner
- Self-employed full time
- Part-time employment

H04: Which of the following options best describes your current position regarding housing or accommodation?

- I live in my own home
- I live in a rented home
- I live with my family
- With my partner in his/her home

- I live with friends
- Refusal to answer

H05: Which of the following options best describes the area where you currently live?

- Center of or in the vicinity of the city center
- Outskirts/Suburbs of the city
- Town or its outskirts/suburbs of a small town
- Village/Countryside
- Center of or in the vicinity of a small town

H11: In which of these regions do you currently live?

- Bratislavský region
- Trnavský region
- Nitriansky region
- Trenčiansky region
- Košický region
- Prešovský region
- Žilinský Region

H12: At which HIV centre are you treated?

- Bratislava - Hospital of Academician Ladislav Dérer - Center for HIV/AIDS Patients I, II
- Praha - Na Bulovce Hospital - HIV/AIDS Center
- Bratislava center
- Praha - Central Military Hospital - HIV center
- Brno - FN Brno - HIV center
- Martin - UN Martin - Outpatient clinic for treatment and dispensary of HIV/AIDS positive people
- Nitra - FN Nitra - Outpatient clinic for HIV
- Banská Bystrica - FNsP FDR - Infectious Diseases Outpatient Clinic I
- Prague (center not specified)

B. List of participating HIV centres and physicians

1. Department of Infectology and Geographical Medicine, Center for Treatment of HIV/AIDS Patients, Academic L. Dérer's University Hospital, Bratislava, Slovakia - Outpatient office I, II

Lubomir Sojak, MD; Pavlina Bukovinova, MD, PhD, MPH

2. Department of Infectology and Geographical Medicine, Center for Management and Treatment of HIV, L Pasteur University Hospital and PJ Safarik University, Košice, Slovakia

Lenka Balogova, MD, PhD; Prof Pavol Jarcuska, MD, PhD

3. Department of Infectology and Travel Medicine, University Hospital, Martin, Slovakia

Assoc. prof. Katarina Simekova, MD, PhD

4. Infectology Clinic FSVaZ by UKF, Faculty Hospital, Nitra, Slovakia

Lubica Piesecka, MD, PhD; Veronika Vahalova, MD

5. Center for Dispensarisation and Treatment of HIV Positive Persons, Department of Infectology, FD Rosevelt Hospital, Banská Bystrica, Slovakia

Diana Volosinova, MD, PhD; Maria Vachalikova, MD

C. Supplementary figures

Figure 1 Treatment-related responses: **(A)** respondent opinions about their HIV treatment (n=80), **(B)** reasons for HIV treatment change (n=32), **(C)** respondent concerns over receiving their HIV treatment during the past 4 weeks (n=80), and **(D)** respondent opinions on the decision-making process regarding their HIV treatment (n=94). HIV, human immunodeficiency virus.

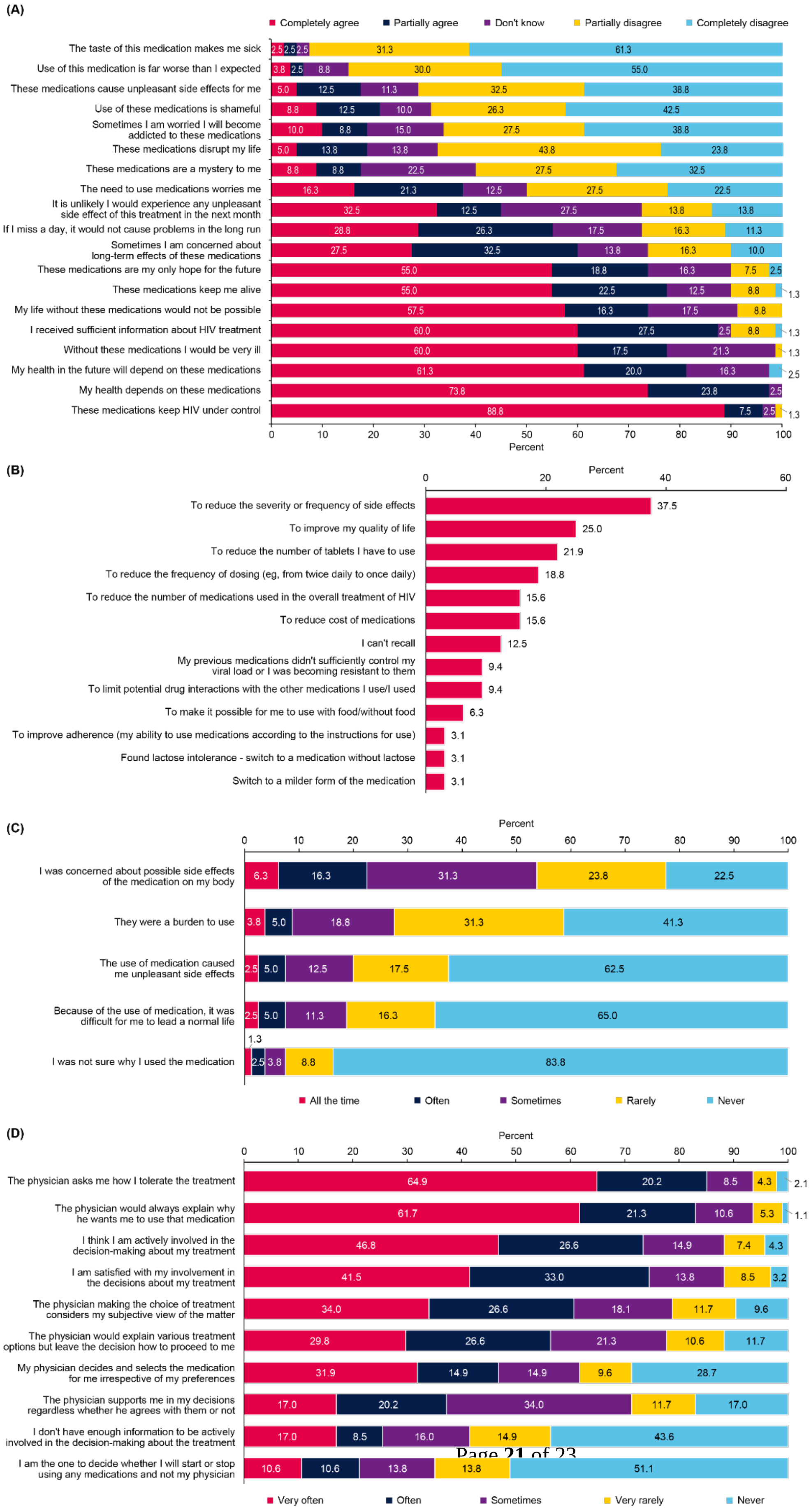

(A)
Completely agree
Partially agree
Don't know
Partially disagree
Completely disagree
The taste of this medication makes me sick 2.5 2.5 2.5 31.3 61.3
Use of this medication is far worse than I expected 3.8 2.5 8.8 30.0 55.0
These medications cause unpleasant side effects for me 5.0 12.5 11.3 32.5 38.8
Use of these medications is shameful 8.8 12.5 10.0 26.3 42.5
Sometimes I am worried I will become addicted to these medications 10.0 8.8 15.0 27.5 38.8
These medications disrupt my life 5.0 13.8 13.8 43.8 23.8
These medications are a mystery to me 8.8 8.8 22.5 27.5 32.5
The need to use medications worries me 16.3 21.3 12.5 27.5 22.5
It is unlikely I would experience any unpleasant side effect of this treatment in the next month 32.5 12.5 27.5 13.8 13.8
If I miss a day, it would not cause problems in the long run 28.8 26.3 17.5 16.3 11.3
Sometimes I am concerned about long-term effects of these medications 27.5 32.5 13.8 16.3 10.0
These medications are my only hope for the future 55.0 18.8 16.3 7.5 2.5
These medications keep me alive 55.0 22.5 12.5 8.8 1.3
My life without these medications would not be possible 57.5 16.3 17.5 8.8
I received sufficient information about HIV treatment 60.0 27.5 2.5 8.8 1.3
Without these medications I would be very ill 60.0 17.5 21.3 1.3
My health in the future will depend on these medications 61.3 20.0 16.3 2.5
My health depends on these medications 73.8 23.8 2.5
These medications keep HIV under control 88.8 7.5 2.5 1.3
0 10 20 30 40 50 60 70 80 90 100
Percent
(B)
Percent
0 20 40 60
To reduce the severity or frequency of side effects 37.5
To improve my quality of life 25.0
To reduce the number of tablets I have to use 21.9
To reduce the frequency of dosing (eg, from twice daily to once daily) 18.8
To reduce the number of medications used in the overall treatment of HIV 15.6
To reduce cost of medications 15.6
I can't recall 12.5
My previous medications didn't sufficiently control my viral load or I was becoming resistant to them 9.4
To limit potential drug interactions with the other medications I use/I used 9.4
To make it possible for me to use with food/without food 6.3
To improve adherence (my ability to use medications according to the instructions for use) 3.1
Found lactose intolerance - switch to a medication without lactose 3.1
Switch to a milder form of the medication 3.1
(C)
Percent
0 10 20 30 40 50 60 70 80 90 100
I was concerned about possible side effects of the medication on my body 6.3 16.3 31.3 23.8 22.5
They were a burden to use 3.8 5.0 18.8 31.3 41.3
The use of medication caused me unpleasant side effects 2.5 5.0 12.5 17.5 62.5
Because of the use of medication, it was difficult for me to lead a normal life 2.5 5.0 11.3 16.3 65.0
I was not sure why I used the medication 1.3 2.5 3.8 8.8 83.8
All the time
Often
Sometimes
Rarely
Never
(D)
Percent
0 10 20 30 40 50 60 70 80 90 100
The physician asks me how I tolerate the treatment 64.9 20.2 8.5 4.3 2.1
The physician would always explain why he wants me to use that medication 61.7 21.3 10.6 5.3 1.1
I think I am actively involved in the decision-making about my treatment 46.8 26.6 14.9 7.4 4.3
I am satisfied with my involvement in the decisions about my treatment 41.5 33.0 13.8 8.5 3.2
The physician making the choice of treatment considers my subjective view of the matter 34.0 26.6 18.1 11.7 9.6
The physician would explain various treatment options but leave the decision how to proceed to me 29.8 26.6 21.3 10.6 11.7
My physician decides and selects the medication for me irrespective of my preferences 31.9 14.9 14.9 9.6 28.7
The physician supports me in my decisions regardless whether he agrees with them or not 17.0 20.2 34.0 11.7 17.0
I don't have enough information to be actively involved in the decision-making about the treatment 17.0 8.5 16.0 14.9 43.6
I am the one to decide whether I will start or stop using any medications and not my physician 10.6 10.6 13.8 13.8 51.1
Very often
Often
Sometimes
Very rarely
Never

Figure 2 Responses related to emotional support: **(A)** emotional support provider at disease diagnosis and at present (n=104) and **(B)** emotional support by partner (n=37). GP, general practitioner; HCP, healthcare provider; HIV, human immunodeficiency virus; PLHIV, person(s) living with HIV.

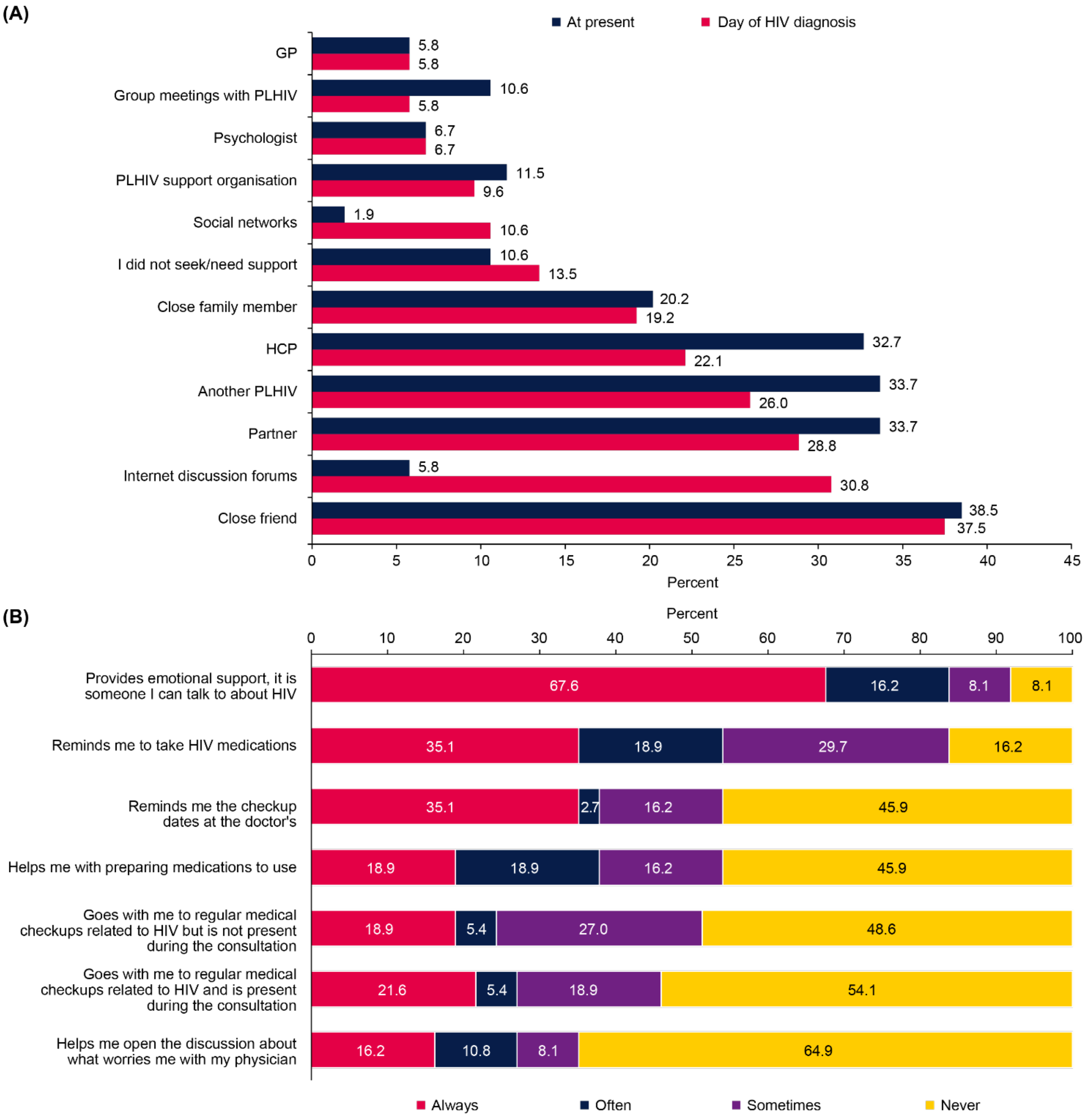

Figure 3 Responses related to stigma experiences: **(A)** afraid to inform of their HIV status in the past 4 weeks (n=98), **(B)** feeling they were being stigmatised in the past 12 months (n=31), and **(C)** populations in need of education regarding HIV. HIV, human immunodeficiency virus.

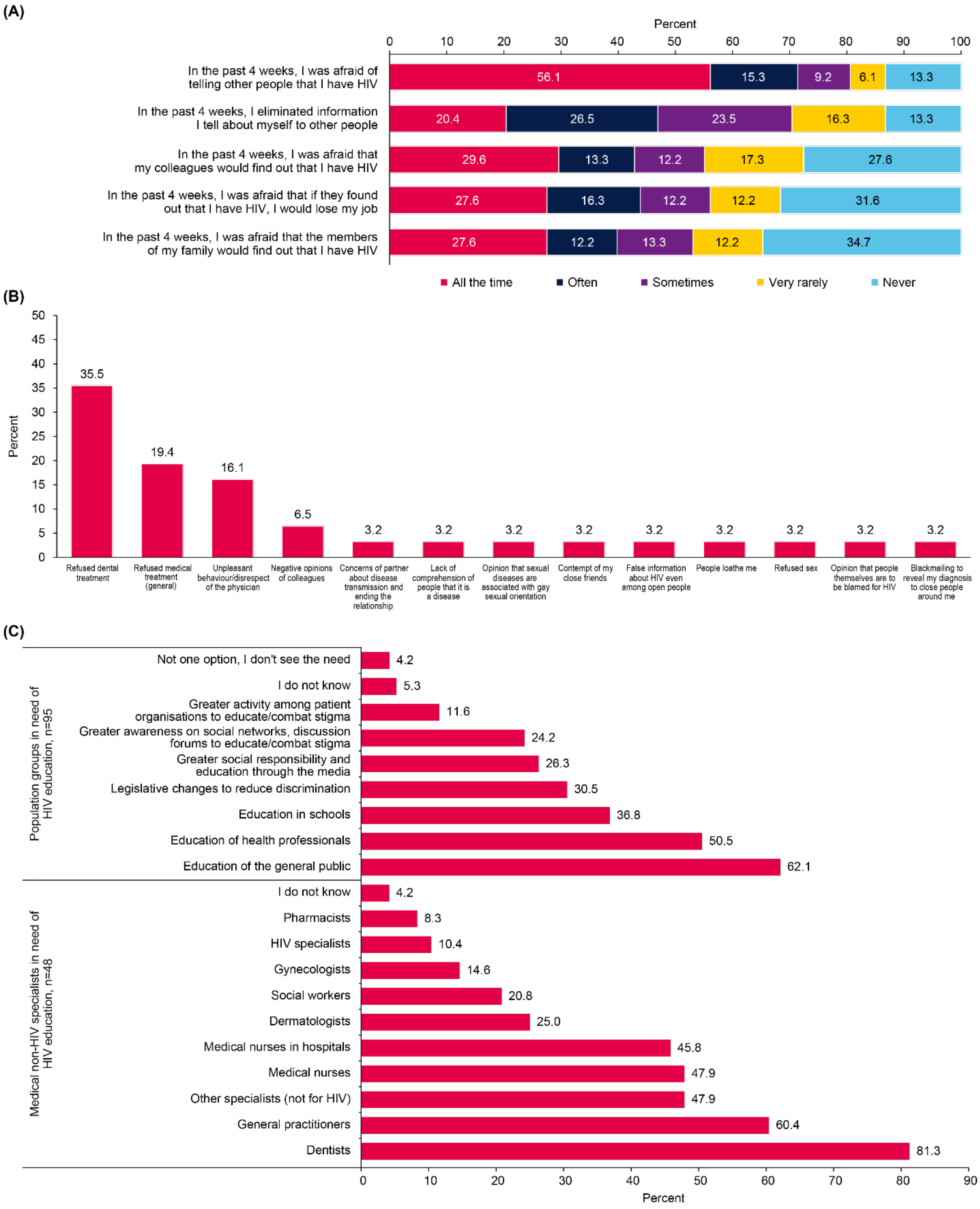

Supplement: Supplementary file 1 [file DataSheet1.PDF]
